# Need for Couple's Awareness About Sexual Health in COVID-19 Pandemic

**DOI:** 10.5195/cajgh.2020.490

**Published:** 2020-03-31

**Authors:** Farzaneh Babapour, Fatemeh Hamidi, Zeinab Hamzehgardeshi

**Affiliations:** 1 Department of Midwifery, Nasibeh School of Nursing and Midwifery, Mazandaran University of Medical Sciences, Sari, Iran; 2 Sexual and Reproductive Health Research Center, Department of Reproductive Health and Midwifery, School of Nursing and Midwifery, Mazandaran University of Medical Sciences, Sari, Iran

In December 2019, an outbreak of pneumonia was identified in Wuhan, China. This novel viral outbreak, now named Coronavirus Disease 2019 (COVID-19), has since quickly spread around the world. It is recognized as a major public health concern and was declared a pandemic disease by the World Health Organization (WHO) on 11 March 2020.^1^ There are similarities to the Severe Acute Respiratory Syndrome (SARS) outbreak in 2003 and the Middle East Respiratory Syndrome (MERS) outbreak in 2012, suggesting that the SARS-CoV-2 virus primarily targets the respiratory system and is transmitted via air droplets and physical contact. Patients were evaluated for viral pneumonia through the special testing utilizing whole-genome sequencing, cell cultures, and polymerase chain reaction (PCR). The virus was recognized as genus beta coronavirus and isolated from biologic samples, placing it alongside SARS and MERS.^2^

The lives of millions of people around the world are currently being affected by the crisis caused by the outbreak of COVID-19. A less obvious repercussion of this pandemic is the potential implications for sexual health among those affected by the pandemic. According to the WHO definition, “sexual health is the state of physical, mental and social well-being in regard to sexuality. It requires a positive and respectful approach to sexuality and sexual relationships, in addition because the possibility of getting pleasurable and safe sexual experiences, freed from coercion, discrimination and violence.”^3^

In developing countries in particular, the provision of sexual and reproductive healthcare services may be negatively impacted during the pandemic.^4^ The COVID-19 pandemic can pose a threat to sexual and reproductive health in both men and women through decreased access to medical care, lack of access condoms, increased incidence of sexually transmitted diseases, etc.^5^ With the global expansion of the COVID-19, watching porn sites has increased. According to Pornhub, pornography use in several countries increased, with global traffic increasing over 11% from late February to March 17, 2020.^6^ Related concerns include the potential increase of gender-based violence, domestic abuse and effects of stigma and discrimination associated with COVID-19.^7^

There are 27 known viruses associated with viremia in human semen, though there is still little known about SARS-CoV-2 in semen. One study has reported that SARS-CoV-2 can be present in the semen of patients with COVID-19, and SARS-CoV-2 may still be detected in the semen of recovering patients.^8^ Data from another study, however, showed that men with COVID-19, both the acute phase and the recovery, of SARS-CoV-2 is absent in samples from both semen and testes.^9^

In addition, direct contact with saliva can potentially transmit the virus, considering the evidence that COVID-19 is a respiratory illness. Other possible transmission routes of SARS-CoV-2 via body fluids include bronchoalveolar-lavage, saliva, blood, urine, feces, sputum, tears.^10^ Therefore, it is important for the general public to know the basics of safe sex to prevent the spread of the COVID-19 virus and still enjoy intimate relationships. Given the importance of continued sexual health during the COVID-19 pandemic, studies of viruses found in semen, especially concerning SARS-CoV-2 need to be a focus of future research.

If future studies were to provide evidence that SARS-CoV-2 can be sexually transmitted, safe sex will be an important part of preventing transmission, through emphasis of transmission prevention methods such as condoms. Community participation, attention to sexual health across all age groups, non-discrimination, access to quality services and information and collaboration will advance the achievement of improved health during the pandemic. Further study is needed due to the importance of this area and lack of existing research about sexual health in COVID-19.
